# Postoperative Rebleeding: The Sword of Damocles in Minimally Invasive Surgery for Intracerebral Hemorrhage

**DOI:** 10.34133/research.1083

**Published:** 2026-01-21

**Authors:** Chuan Wang, Ruchong Fan, Zi Lin, Shiling Chen, Chao Pan, Hao Nie, Chuan Qin, Xuan Wu, Zhouping Tang

**Affiliations:** ^1^ Department of Neurology, Tongji Hospital, Tongji Medical College, Huazhong University of Science and Technology, Wuhan, Hubei 430030, P. R. China.; ^2^ Department of Geriatrics, Tongji Hospital, Tongji Medical College, Huazhong University of Science and Technology, Wuhan, Hubei 430030, P. R. China.; ^3^ Hubei Key Laboratory of Neural Injury and Functional Reconstruction, Huazhong University of Science and Technology, Wuhan, Hubei 430030, P. R. China.; ^4^ Key Laboratory of Vascular Aging, Ministry of Education, Tongji Hospital, Tongji Medical College, Huazhong University of Science and Technology, 1095 Jiefang Avenue, Wuhan, Hubei 430030, P. R. China.; ^5^ State Key Laboratory for Diagnosis and Treatment of Severe Zoonotic Infectious Diseases, Wuhan, Hubei 430030, P. R. China.

## Abstract

Intracranial hemorrhage (ICH) represents a subtype of stroke characterized by increased mortality and disability rates. Early evacuation of the hematoma is essential for the effective management of ICH. Currently, minimally invasive surgery (MIS) has emerged as a promising alternative to traditional craniotomy by offering advantages, such as reduced operating time, minimal surgical trauma, and accelerated recovery. Nonetheless, postoperative rebleeding remains an important complication that adversely affects the functional outcomes and survival rates of the affected patients. Thus, acquiring a thorough understanding of postoperative rebleeding following MIS for ICH is crucial for enhancing neurological functional outcomes. This review aimed to synthesize current evidence regarding the definition of postoperative rebleeding following MIS for ICH, elucidate the mechanisms contributing to this phenomenon, and identify the associated risk factors, including both surgical and patient-related factors. In addition, the review discusses contemporary strategies for the prevention and management of postoperative rebleeding. Furthermore, it explores prospective advances in dynamic risk prediction and early detection of postoperative rebleeding using artificial intelligence and real-time biosensors. This review offers a reference for clinical practice and guides future studies aimed at reducing the risk of postoperative rebleeding after MIS for ICH, thereby enhancing the outcomes of patients.

## Introduction

Intracerebral hemorrhage (ICH) refers to an acute cerebrovascular disease caused by the rupture of cerebral blood vessels, resulting in blood accumulation in the brain parenchyma. Characterized by high incidence, high disability rate, and high mortality, it significantly undermines public health. From an etiological perspective, ICH is primarily categorized into spontaneous ICH (accounting for approximately 80% of cases, often caused by spontaneous rupture of small arteries due to hypertension) and secondary ICH (caused by trauma, vascular malformations, tumors, coagulation disorders, thrombolytic therapy, anticoagulant therapy, etc.) [1]. This review primarily examines recent findings on the serious complication of rebleeding following minimally invasive surgery (MIS) in patients with spontaneous ICH. In addition, considering the growing number of patients currently taking anticoagulants for underlying conditions such as atrial fibrillation, we also explored issues related to postoperative rebleeding in anticoagulant-related ICH in the “Patient-related factors of postoperative rebleeding” section.

Spontaneous ICH represents a severe form of cerebrovascular disease, predominantly caused by nontraumatic rupture of blood vessels in the brain parenchyma [2]. It accounts for 10% to 15% of all stroke cases and is associated with the highest mortality and disability rates among stroke subtypes [3]. Its 30-d mortality rate ranges from 35% to 52% [4]. Approximately 20% of patients regain independence in daily activities after 6 months, and the overall prognosis remains generally poor [4–6]. Furthermore, the pathophysiological mechanisms underlying neuronal death in ICH can be primarily attributed to the mass effect of the intracerebral hematoma and the release of neurotoxic components. Prolonged persistence of the hematoma in the cranial cavity correlates with a worse prognosis [7]. Therefore, early evacuation of the hematoma is essential for the effective management of ICH [8–10].

Hematoma evacuation strategies for ICH predominantly encompass traditional craniotomy and minimally invasive hematoma evacuation procedures [6]. Although traditional craniotomy can help effectively remove hematoma, its clinical application is limited because of substantial trauma, the high rates of associated complications, and prolonged postoperative recovery [9]. Recent progress in minimally invasive hematoma evacuation techniques has opened new avenues for the management of ICH. The advantages of MIS include shorter length of surgery, accelerated postoperative recovery, and minimal trauma to adjacent brain tissues, collectively decreasing the incidence of postoperative complications [11–13]. Furthermore, MIS has been linked to a significant decrease in postoperative mortality rates [9,14]. Despite these benefits in minimizing postoperative complications, certain risks persist, including postoperative rebleeding, infection, residual hematoma, cerebral edema, and neurological dysfunction [15,16]. Among these complications, postoperative rebleeding is particularly concerning, as it constitutes one of the most severe surgical complications and frequently results in mortality [6,17–24]. For instance, Song et al. [25] reported postoperative mortality rates of 7.02% in patients experiencing rebleeding following MIS, compared to 0.84% in those who did not. Moreover, postoperative rebleeding may exacerbate postoperative complications, such as infection and cerebral edema, undermining patient recovery and quality of life [26,27]. Notably, MIS also reduces the risk of postoperative rebleeding compared to traditional craniotomy. Several meta-analyses and randomized trials have reported lower rebleeding rates following MIS [28,29]. A systematic review and meta-analysis reported that in patients with spontaneous ICH, MIS lowers rebleeding rates compared to traditional craniotomy (odds ratio, 0.42; 95% confidence interval, 0.28 to 0.64) [30]. Although accumulating evidence indicates that the overall risk of rebleeding is significantly lower with MIS than with craniotomy, this finding does not imply that rebleeding is completely addressed in MIS. On the contrary, the rapid advancement and widespread adoption of MIS necessitate in-depth studies on postoperative rebleeding.

**Fig. 1.**
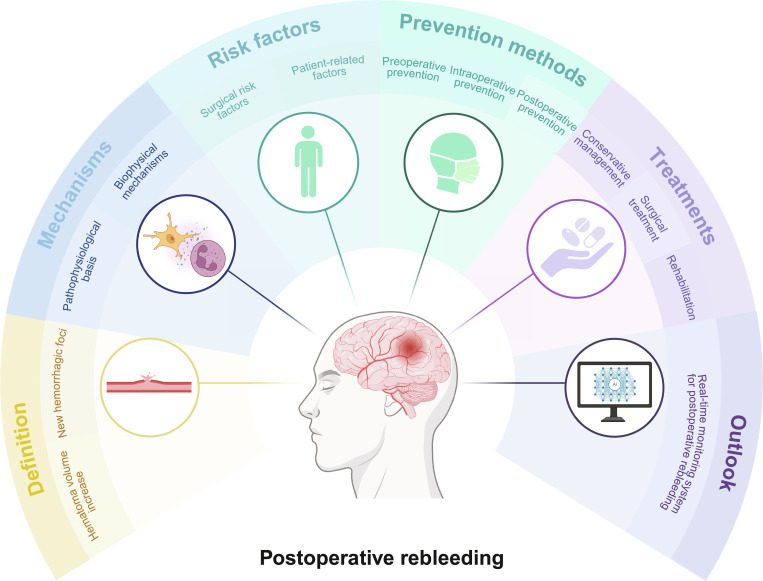
General overview of postoperative rebleeding. Created in BioRender.

Understanding the mechanisms underlying postoperative rebleeding following MIS in patients with spontaneous ICH, the early identification of rebleeding, and the characterization of risk factors associated with poor prognosis are crucial for enhancing neurological outcomes. This review addresses the definition of postoperative rebleeding following MIS, elucidates the underlying mechanisms, and identifies the risk factors of rebleeding, encompassing both surgical and patient-related factors. In addition, it discusses preventive and therapeutic strategies. The review also delineates future research directions concerning postoperative rebleeding in the context of MIS for ICH and proposes an ideal model for real-time monitoring of postoperative rebleeding. This review aimed to guide clinical practice and future studies attempting to mitigate the risk of postoperative rebleeding following MIS for ICH, thereby improving patients’ outcomes. General overview of postoperative rebleeding is shown in Fig. 1.

## Definition of Postoperative Rebleeding

Postoperative rebleeding is a key end-point event for evaluating the efficacy of MIS for ICH, with its definition requiring a comprehensive assessment of multidimensional information, including imaging and clinical manifestations. Currently, definitions vary across studies but mainly rely on postoperative imaging.

**Fig. 2.**
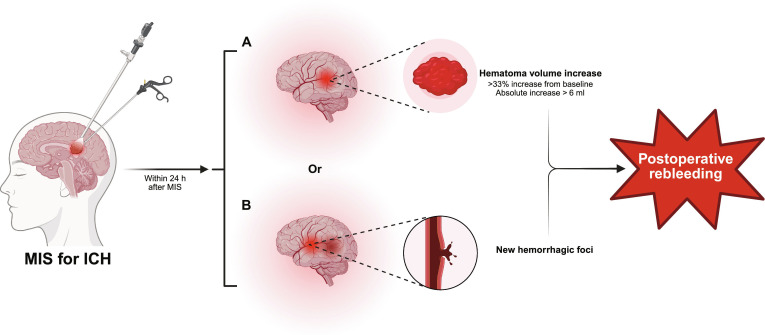
Definition of postoperative rebleeding. Within 24 h of MIS for intracerebral hemorrhage, (A) an increase in hematoma volume (exceeding 33% of preoperative volume or an absolute increase of more than 6 ml) or (B) the emergence of new hemorrhagic foci constitutes postoperative rebleeding. Created in BioRender.

Imaging assessment serves as the objective foundation for definition, typically confirmed by postoperative computed tomography (CT) or magnetic resonance imaging, which shows increased volume of hematoma or the emergence of new hemorrhagic foci [23,24,31–33]. To standardize the criteria, we recommend adopting quantitative thresholds proposed by the American Heart Association/American Stroke Association (AHA/ASA) guidelines. A postoperative increase in the volume of hematoma of more than 33% of preoperative volume or an absolute volume increase exceeding 6 ml within 24 h, or the presence of new hemorrhagic foci, constitutes a reliable diagnostic standard for rebleeding (Fig. 2) [34,35]. Clinically, postoperative neurological deterioration (e.g., decreased level of consciousness and limb weakness) serves as an important warning sign but requires careful differentiation. Deterioration associated with rebleeding typically exhibits a close temporal relationship with radiologically confirmed hemorrhagic events, necessitating systematic exclusion of neurological decline caused by other postoperative complications [32,33,36,37]. For instance, neurological deterioration caused by surgical trauma or cerebral edema may progress slowly, showing no evidence of active hemorrhage on imaging but exhibiting widened edema bands or increased mass effect [38]. Intracranial infection or hydrocephalus is typically accompanied by specific symptoms, such as fever and meningeal irritation signs, and can be differentiated by head CT or lumbar puncture [39]. Therefore, diagnosing rebleeding necessitates the integration of clinical manifestations of neurological deterioration with immediate imaging findings.

In summary, defining rebleeding after MIS for ICH requires the integration of objective quantitative imaging criteria with careful differentiation of clinical symptoms. We recommend prioritizing the use of the aforementioned AHA/ASA imaging quantification standards in future studies to enhance comparability between research findings and the clinical relevance of conclusions.

## Mechanisms Underlying Postoperative Rebleeding

**Fig. 3.**
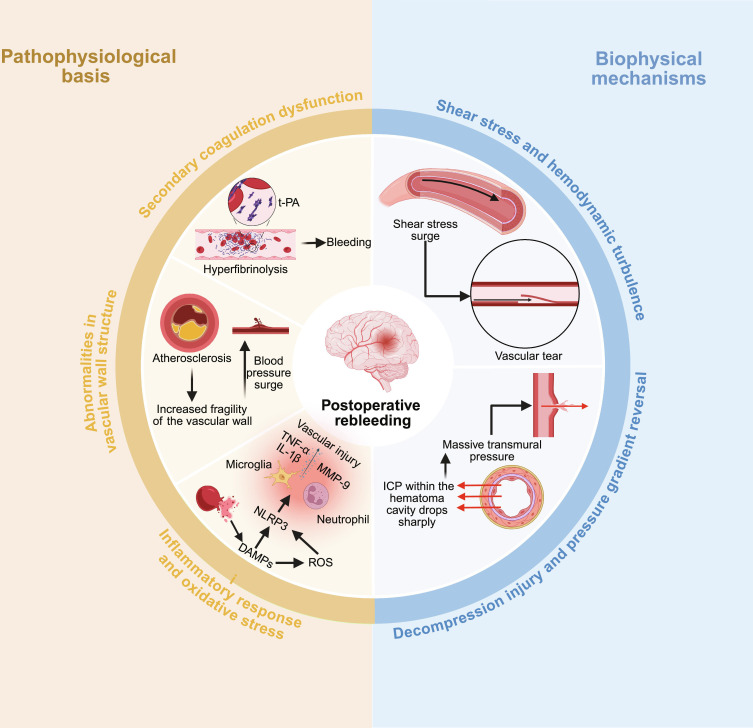
Mechanisms of postoperative rebleeding. Pathophysiological basis (left). Biophysical mechanism (right). Surgical evacuation disrupts the hemostatic clot, potentially inducing hyperfibrinolysis (t-PA), while preexisting vascular fragility and a surgery-aggravated inflammatory response (involving MMP-9, ROS, and TNF-α/IL-1β) compromise vascular integrity. Concurrently, rapid decompression induces a critical shear stress surge upon reperfusing vessels and reverses the pressure gradient across the hematoma cavity wall, collectively promoting the rupture of vulnerable vessels and leading to rebleeding. Created in BioRender.

Although MIS significantly reduces the trauma associated with traditional craniotomy, as an interventional procedure, it may disrupt the established pathophysiological balance in the cranium, thereby inducing or exacerbating rebleeding [40,41]. The mechanism underlying this complication is complex, involving patient-specific factors, hematoma characteristics, and surgical manipulation. It can be elucidated at 2 levels, including the pathophysiological basis and the biophysical mechanisms (Fig. 3).

### Pathophysiological basis

Postoperative rebleeding occurs because the hematoma cavity and surrounding areas are in an extremely unstable and fragile state following primary hemorrhage and secondary to surgical trauma [42,43]. They impair hemostasis, lead to secondary coagulation dysfunction, abnormal vascular wall structure or direct injury, and exacerbate inflammatory responses and oxidative stress.

#### Imbalance in hemostatic mechanisms and secondary coagulation dysfunction

The brain tissue is rich in tissue factor, which initiates the extrinsic coagulation pathway after primary hemorrhage, forming unstable blood clots within hematomas [44]. Minimally invasive puncture procedures for hematoma evacuation remove the core clot that provides “autologous packing”, disrupting the natural hemostatic barrier and increasing the risk of rebleeding. Fu et al. [43] indicated that MIS disrupts the packing effect of clots and can lead to a postoperative rebleeding rate as high as 14.3%. Furthermore, intraoperative and postoperative saline irrigation for maintaining drainage patency not only dilutes local coagulation but also activates the fibrinolytic system (e.g., tissue-type plasminogen activator [t-PA]), triggering hyperfibrinolysis. This dissolves residual fragile thrombi, reexposing ruptured vessels and leading to rebleeding. Li et al. [45] also confirmed the correlation between intraoperative fibrinolytic system activation and rebleeding. Moreover, extensive hematoma absorption following ICH can induce consumptive coagulopathy, while surgical trauma exacerbates local and systemic hemostatic dysfunction [46]. Thus, abnormal minimally invasive surgical hemostasis and secondary coagulopathy represent key mechanisms underlying rebleeding.

#### Abnormalities in vascular wall structure

Patients with hypertensive ICH often present with underlying vascular diseases, such as small-artery atherosclerosis, lipid hyalinosis, or microaneurysms, leading to structural abnormalities and increased fragility of the vascular wall [47]. In the acute postoperative period after MIS, when blood pressure fluctuates markedly, the affected vessels cannot withstand sudden pressure surges [48]. Furthermore, surgical instruments may directly damage blood vessels within the access pathway, including both normal and diseased vessels, during puncture or catheter placement. Even without causing macroscopically visible injury to large vessels, microscopic disruption of the capillary network may serve as a source of rebleeding [49].

#### Exacerbation of inflammatory responses and oxidative stress

Following ICH, components of the hematoma, such as hemoglobin and iron ions released from lysed red blood cells, serve as potent damage-associated molecular patterns, triggering local inflammatory responses and oxidative stress. Activation of the NLRP3 (NLR family pyrin domain containing 3) inflammasome is a key upstream event in this process. Hemoglobin and iron ions activate the NLRP3 inflammasome in microglia, leading to the maturation and release of proinflammatory cytokines, such as interleukin-1β (IL-1β), via a caspase-1-dependent pathway [50]. The high amount of reactive oxygen species (ROS) generated by iron-catalyzed Fenton reactions also activates the NLRP3 inflammasome, forming a positive feedback loop that exacerbates inflammation [51,52]. Downstream manifestations include sustained microglial activation, neutrophil infiltration, and massive release of matrix metalloproteinases (e.g., MMP-9) and inflammatory mediators (tumor necrosis factor-α [TNF-α] and IL-1β) [53–55]. MMP-9 degrades extracellular matrix and vascular basement membrane components, compromising the integrity of vascular structure [56]. As a type of secondary injury, MIS significantly potentiates these pathological responses at multiple levels (e.g., by exacerbating iron overload and oxidative stress), forming a vicious cycle that undermines vascular stability and promotes rebleeding [57]. Crucially, elucidating this cascade reveals promising therapeutic targets. Recent preclinical studies have shown that pharmacological inhibition of the NLRP3 inflammasome (e.g., with MCC950) or chelation of free iron can effectively suppress neuroinflammation, lower MMP-9 expression, and improve the outcomes of ICH, bridging mechanistic understanding to potential future interventions [58,59].

### Biophysical mechanisms

Beyond pathophysiological mechanisms, the biophysical effects of minimally invasive decompression procedures are also key factors in inducing rebleeding. Although surgical decompression remains the core therapeutic approach, the abrupt changes in pressure may elevate the risk of shear stress and lead to decompression injury and vascular rupture.

#### Shear stress and hemodynamic turbulence

Shear stress is the tangential force generated parallel to the vessel wall as blood flows along it, typically remaining stable within physiological ranges [60]. Evacuation of hematoma through MIS suddenly decompresses the previously compressed and collapsed capillaries and arterioles and reopens their lumens. The autoregulatory function of these vessels, long subjected to hypoperfusion, is severely compromised [61]. When suddenly exposed to a rapid increase in blood flow perfusion and intraluminal pressure, the shear stress on the vessel wall abruptly increases, exceeding its tolerance in the pathological state [62]. This abrupt increase in shear stress can directly tear the structurally fragile vessel wall (especially in diseased vessels), leading to rebleeding.

#### Decompression injury and pressure gradient reversal

Decompression injury represents the most characteristic biophysical mechanism of rebleeding following MIS for ICH. After the incidence of hemorrhage, the high-pressure cavity formed by the intracranial hematoma compresses surrounding tissues while simultaneously leading to “compression hemostasis” on the bleeding site [38,63,64]. Intracranial pressure (ICP) within the hematoma cavity drops sharply following minimally invasive aspiration of the hematoma. However, pressure reduction in the surrounding brain tissue lags due to edema and other factors, reversing the pressure gradient. The pressure is higher in the surrounding tissues than in the hematoma cavity [65]. This reversal forms a massive transmural pressure difference that acts on the vascular stumps and fragile vessel segments within the hematoma cavity wall. This pressure reopens previously compressed and closed bleeding sites or causes pathological vascular dilatation and rupture, triggering rebleeding [37,66].

In summary, surgical manipulation biophysically alters the preexisting cerebral vasculature, exacerbating the existing pathophysiological processes and establishing a vicious cycle that leads to rebleeding. A thorough understanding of this mechanism is crucial for developing strategies to prevent and manage postoperative rebleeding.

## Risk Factors of Postoperative Rebleeding

**Fig. 4.**
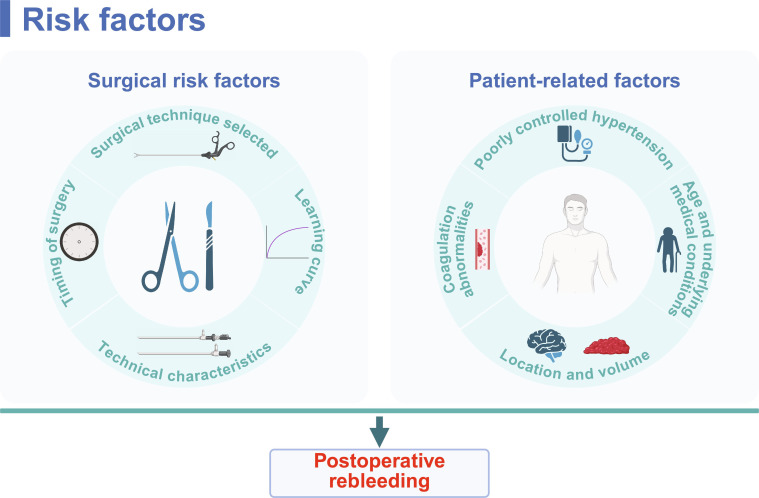
Risk factors of postoperative rebleeding. Surgical risk factors (left): the specific minimally invasive technique employed, surgical timing, and the operator’s learning curve. Patient-related factors (right): poorly controlled hypertension, hematoma location and volume, coagulation abnormalities, as well as advanced age (>75 years) and comorbidities. The combined influence of these factors determines the risk of rebleeding, highlighting the need for individualized assessment and comprehensive management in clinical practice. Created in BioRender.

To effectively mitigate the risk of rebleeding, it is essential to identify and manage its risk factors after MIS. On the basis of relevant studies, we summarized the principal risk factors associated with rebleeding following MIS. These factors can be categorized into 2 distinct types: surgical factors and patient-related factors (Fig. 4).

### Surgical risk factors for postoperative rebleeding

The primary and most important risk factor for postoperative rebleeding is the choice of surgical technique. The available minimally invasive treatments for ICH primarily include stereotactic aspiration with thrombolysis (SAT), endoscopic surgery (ES), and minimally invasive parafascicular surgery (MIPS). Previous studies have shown that the surgical technique selected for MIS can significantly affect the rate of postoperative rebleeding [67,68].

SAT is currently the most extensively studied type of MIS, suitable for deep hematomas, such as basal ganglia hemorrhages. This technique uses stereotactic guidance for precise needle placement, followed by catheter-assisted aspiration combined with thrombolytic agents (e.g., urokinase), to promote dissolution and drainage of residual hematoma. It offers several advantages, including minimal trauma (requiring only a 2- to 3-mm burr hole), short operative time, and the possibility of local anesthesia [69–73]. The Minimally Invasive Surgery Plus Alteplase for Intracerebral Hemorrhage Evacuation (MISTIE) II and III series evaluated the efficacy and safety of SAT for treating moderate-to-large ICH (30 to 100 ml). MISTIE II reported symptomatic rebleeding rates of 5% to 8%, predominantly associated with postoperative fluctuations in blood pressure (systolic > 160 mmHg) and use of thrombolytic drugs (e.g., alteplase dosage and frequency) [74]. Following further refinement of the thrombolytic protocol in MISTIE III, the rebleeding rate decreased to 4% to 6%. These results underscore the importance of precise puncture and appropriate timing of thrombolysis (resolving the need for too early administration within 6 h) for reducing the risk of hemorrhage [75]. ES, clinically applied since the 1980s, enables direct visualization for hematoma clearance and hemostasis, typically achieving evacuation rates of 80% to 95% [49,76]. A meta-analysis by Minimally Invasive Surgeries for Spontaneous Hypertensive Intracerebral Hemorrhage (MISICH) et al. showed a rebleeding rate of 5.6% to 10.3% in the endoscopic group, potentially attributable to incomplete intraoperative hemostasis (particularly for arterial bleeding) and inadequate exploration of the hematoma cavity [77]. MIPS is a tract-based technique utilizing a paracisternal rather than a cortical approach. By preserving white matter tracts, it is useful for lobar and anterior basal ganglia hemorrhages, emphasizing the importance of early intervention (<24 h) and complete hematoma evacuation [78–80]. The Early Minimally Invasive Removal of Intracerebral Hemorrhage (ENRICH) trial reported a rebleeding rate of only 3.3% in the MIPS group, significantly lower than that in the medical management group (*P* < 0.05) [23]. This low rebleeding rate may be related to the pathway design, thorough hematoma evacuation, and intraoperative image guidance. However, the learning curve for this technique is steep, and operator experience is particularly crucial for preventing hemorrhagic complications. Each of these 3 minimally invasive surgical procedures exhibits unique characteristics. A summary of the principal technical features of these procedures is provided in Table 1.

**Table 1.** Comparison of key technical features of 3 minimally invasive surgeries

MIS	Guidance method	Evacuation mechanism	Instrument size (mm)	Main advantage	Technical characteristics linked to rebleeding risk	Reference
SAT	Stereotactic navigation	Aspiration + thrombolysis	4.8	Minimal trauma, deep hematoma suitable	Nonvisualized operation (“blind”), influence of thrombolytic drugs	[69–73,76]
ES	Endoscopic visualization	Direct visualization + suction	10.0	High clearance rate, direct visualization for hemostasis	Limited field of view (blind spots), restricted instrument maneuverability	[49,76]
MIPS	Tubular navigation	Intraluminal suction	15.8	White matter protection, early evacuation	Potential vascular injury risk from larger channel diameter	[76,78–80]

In addition to procedural differences, the timing of surgery significantly affects the risk of rebleeding [81]. While early surgery (<6 to 8 h) facilitates rapid decompression, it may increase rebleeding risk due to the formation of unstable thrombi [82]. In the ENRICH trial, the MIPS group had a median time from onset to surgery of 16.8 h. This relatively late time window may partly explain its lower rebleeding rate. Conversely, SAT is often conducted at a later time point (e.g., median 58 h in MISTIE III), but thrombolysis itself introduces an additional bleeding risk. ES offers greater temporal flexibility, although ultra-early (<12 h) procedures increase the risk of rebleeding, particularly with uncontrolled blood pressure. This temporal discrepancy suggests that different procedures may have different optimal timing windows, requiring a balance between early decompression benefits and rebleeding risk.

Beyond timing, the technical characteristics of each minimally invasive approach significantly affect the risk of rebleeding. The specific technical factors contributing to rebleeding with SAT primarily originate from its “blind operation” nature and reliance on thrombolytic agents. First, since the procedure is conducted without direct visualization, the operator cannot directly observe active bleeding sites in the hematoma cavity. In particular, for small arterial bleeds, compression via the puncture site alone may not achieve reliable hemostasis. Second, thrombolytic agents (e.g., alteplase) not only dissolve blood clots but may also act on fresh thrombi forming on unstable ruptured vessel walls, potentially triggering recurrent bleeding. The emphasis on blood pressure control and high-risk time windows (e.g., premature thrombolysis within 6 h after the procedure) in the MISTIE series can help mitigate this inherent technical risk. Therefore, the risk of rebleeding with SAT is closely associated with the precision of puncture and the dose, frequency, and timing of thrombolytic drug [83,84]. Although ES technology provides direct visualization, its rebleeding risk is directly linked to the physical limitations of the endoscope itself. Endoscopes offer a “tubular field of view”, where the lateral walls and corners of the hematoma cavity may fall into blind spots, potentially missing active bleeding points in these areas. Furthermore, the maneuverability and flexibility of endoscopic instruments, such as suction devices and electrocoagulators, are restricted within the confined surgical channel. When managing irregularly shaped or poorly positioned hematomas, these instruments may inadvertently damage surrounding healthy vessels through traction or accidental contact, leading to iatrogenic bleeding. The “insufficient clarity of the surgical field” mentioned in a meta-analysis reflects limited visualization, while “inadequate exploration of the hematoma cavity” is directly associated with blind spots during manipulation. Therefore, the operator’s experience is crucial for detecting blind spots and utilizing angled endoscopes or adjusting the instrument position [85,86]. The lower rebleeding rate associated with the MIPS technique originates from its pathway design and emphasis on thorough debridement; however, its unique technical characteristics also introduce specific risks. MIPS uses a larger-diameter channel (approximately 15.8 mm), which facilitates instrument manipulation and clot removal. However, during needle insertion and catheter placement, particularly when traversing deep brain tissues, blunt tissue separation using such a large channel increases the risk of damage to small perforating arteries along the path. However, the extremely low rebleeding rate observed in the ENRICH trial suggests that this potential risk can be effectively mitigated through thorough intraoperative exploration, hemostasis within the hematoma cavity, and precise image-guided path planning to avoid major vascular areas. This also underscores that the stringent demands this technique places on operator experience and learning curve [87,88]. In summary, the 3 minimally invasive techniques each harbor distinct sources of rebleeding risk due to differences in instrument size, visualization capabilities, and operational mechanisms. Table 2 presents the risk of rebleeding associated with the 3 minimally invasive surgeries as reported in their respective primary randomized controlled trials (RCTs).

**Table 2.** Differences in rebleeding risks in major RCTs for 3 minimally invasive surgeries

RCT study	Technique	Sample size	Rebleeding rate (%)	Independent risk factors for rebleeding	Reference
MISTIE II	SAT	~120	5–8	Blood pressure > 160 mmHg, improper thrombolysis management	[74,83,84]
MISTIE III	SAT	506	4–6	Blood pressure > 160 mmHg, early thrombolysis (<6 h)	[75,83,84]
MISICH	ES	156	5.6–10.3	Inadequate visual field clarity	[77,85,86]
ENRICH	MIPS	300	3.3	Inappropriate surgical approach selection	[23,87,88]

Because of these technical differences, the effects of learning curves vary significantly across techniques. ES and MIPS demand higher levels of operator skill, requiring specialized training and experience accumulation. Studies have shown that ES rebleeding rates markedly decrease from 15% to less than 5% with the increase in operator experience [89,90]. In contrast, the learning curve for the SAT is relatively gentle, although precise puncture localization and appropriate thrombolytic management still need substantial experience [69,91]. The MISTIE II and III trials demonstrated that strict protocol adherence and team training are critical for reducing the rebleeding risk of SAT. This disparity in learning curves explains the significant variation in rebleeding rates among different minimally invasive techniques.

In summary, surgical approach, timing, and operator experience collectively constitute major procedure-related factors affecting rebleeding after MIS. Surgical strategies should be individualized on the basis of each patient’s specific circumstances.

After comprehensively comparing the risk of rebleeding across various techniques, it is crucial to evaluate the quality and limitations of evidence from major RCTs in this field. This facilitates prudent interpretation of the aforementioned data and guides future studies. First, major RCTs exhibited specific limitations. For the MISTIE series evaluating SAT, despite standardized procedural and thrombolytic protocols, variations in operator experience and technical proficiency across different centers may have compromised the homogeneity of rebleeding rate reporting and the generalizability of functional outcomes. For instance, MISTIE III failed to meet its primary end point. Thus, caution is warranted in equating the critical safety end point of rebleeding rate with clinical benefit. For the ENRICH trial demonstrating the advantages of minimally invasive transfissural surgery (MIPS), its extremely low rebleeding rate was due to the presence of a highly specialized research team, raising questions about the scalability of the technique across different levels of healthcare institutions. As an emerging technology, its preliminary conclusions must be validated by independent RCTs. Regarding ES, as covered in the MISICH analysis, the techniques exhibited marked heterogeneity in terms of endoscope models, operative techniques, and clearance criteria. Such variations may cause fluctuations in rebleeding rates across studies, posing challenges for cross-study comparisons and drawing consistent conclusions. Beyond individual trials, methodological challenges are common in this field. Surgical trials face difficulties in implementing blinding, introducing risks of performance and assessment bias. Strict inclusion/exclusion criteria limit the generalizability of findings to broader, more complex clinical populations. In summary, existing RCTs provide high-grade but imperfect evidence for assessing rebleeding risk. When applying such data to clinical decision-making, it is crucial to recognize that they came from controlled, idealized settings. Therefore, individualized strategies should not only reference trial data but also integrate the technical expertise of local teams and the specific circumstances of each patient. Future studies should standardize technical procedures, validating efficacy in real-world settings and exploring imaging or biomarkers capable of accurately predicting individualized risks of rebleeding.

### Patient-related factors of postoperative rebleeding

Beyond surgical factors, patient-related factors significantly affect the risk of rebleeding following MIS.

Poorly controlled hypertension is a major risk factor for postoperative rebleeding [92–94]. Studies have indicated that patients with preoperative hypertension are more prone to rebleeding postoperatively, likely due to increased vascular fragility [95]. In addition, significant fluctuations in intraoperative blood pressure may lead to further vascular damage, thereby increasing the risk of rebleeding [96]. Therefore, strict postoperative blood pressure management is particularly crucial. In a study on spontaneous ICH, poor blood pressure control within 3 months after surgery was significantly associated with increased risk of rebleeding and mortality rates. Conversely, strict blood pressure control significantly reduced the incidence of rebleeding and effectively improved the outcomes of patients [97,98]. Thus, postoperative blood pressure should be closely monitored, and appropriate antihypertensive medications should be used to maintain blood pressure within a safe range [99]. Strict postoperative blood pressure control can reduce rebleeding risk, improve the survival rates of patients, and promote the quality of life [100].

With the aging population and rising prevalence of cardiovascular diseases, an increasingly large population of patients needs long-term anticoagulants, such as warfarin and direct oral anticoagulants (DOACs), due to conditions such as atrial fibrillation, coronary stent implantation, or mechanical heart valve replacement. Therefore, anticoagulant-related ICH is more commonly observed in clinical practice. In patients undergoing MIS, inherent coagulation dysfunction significantly increases the risk of postoperative rebleeding. Therefore, anticoagulant-related ICH has been included in the following discussion. In addition to hypertension, the preoperative medication history of patients, particularly the use of anticoagulants and antiplatelet agents, represents a critical independent risk factor for postoperative rebleeding [101]. These medications directly interfere with the body’s normal coagulation mechanisms, thereby increasing the risk for rebleeding subsequent to surgical trauma. Anticoagulants systemically reduce the body’s clotting potential. MIS itself leads to trauma and vascular damage. Under effective anticoagulation, local clot formation becomes difficult, unstable, or prone to dissolution, leading to persistent bleeding at the surgical site or the formation of new hematomas [102]. Numerous observational studies and meta-analyses have demonstrated a clear association between preoperative use of vitamin K antagonists (e.g., warfarin) or DOACs (e.g., dabigatran, rivaroxaban, and apixaban) and increased risk of postoperative rebleeding [103,104]. Warfarin severely impairs the amplifying effect of the coagulation cascade by inhibiting the synthesis of vitamin K-dependent coagulation factors (II, VII, IX, and X) [105]. DOACs directly inhibit specific coagulation factors (e.g., factor Xa or factor IIa), offering a rapid onset but lacking specific antagonists for swift reversal of their anticoagulant effects [106]. Antiplatelet drugs primarily affect platelet activation and aggregation, critical steps in initial hemostasis. At surgical sites, platelets cannot effectively form a “platelet plug” to seal damaged microvessels, impairing the first step of hemostasis. Patients with spontaneous ICH who are receiving long-term antiplatelet therapy (e.g., aspirin and clopidogrel) for cardiovascular or cerebrovascular diseases suffer from a significantly elevated risk of postoperative rebleeding [107]. One study indicated that while monotherapy with antiplatelet agents increases rebleeding risk compared to anticoagulation therapy, the absolute risk remains lower [108]. This suggests that in certain circumstances, single antiplatelet therapy may be a safer option. However, dual antiplatelet therapy (DAPT) substantially increases the risk of rebleeding under specific conditions. Studies have indicated that DAPT increases the risk of rebleeding compared to single antiplatelet therapy [109,110]. Therefore, potential benefits and risks must be carefully weighed when selecting a treatment regimen. In summary, coagulation abnormalities caused by preoperative treatment with anticoagulants or antiplatelet agents in patients with spontaneous ICH substantially increase the risk of postoperative rebleeding. Therefore, prompt postoperative assessment of coagulation function and implementation of proactive reversal strategies for patients receiving anticoagulants or antiplatelet agents may lower rebleeding risk in such patients.

The location of bleeding and hematoma volume are also closely associated with rebleeding risk. Deep hemorrhages in areas such as the brainstem and basal ganglia increase rebleeding risks due to complex anatomy and challenging surgical manipulation [25,111,112]. In addition, larger hematomas are associated with increased rebleeding risk due to incomplete evacuation [23,24]. Thus, thorough intraoperative evacuation of hematoma is crucial for reducing rebleeding risk [36].

Patient age and underlying medical conditions are also significant factors. Studies have indicated that because of increased vascular fragility and higher prevalence of comorbidities, elderly patients with ICH who are at least 75 years of age may face a greater risk of rebleeding following MIS [113,114]. In addition, underlying conditions, such as hypertension and diabetes, can induce structural changes in blood vessels, further increasing the risk of bleeding [115–117]. Thus, in MIS for ICH, preoperative assessment of patients’ comorbidities and age is critical for reducing the risk of postoperative rebleeding.

Thus, a comprehensive evaluation of patient age, underlying conditions, preoperative medication history, coagulation status, and hemorrhagic characteristics is necessary to establish individualized blood pressure management targets and surgical strategies. This approach most effectively decreases postoperative rebleeding risk and improves overall prognosis.

In summary, reducing postoperative rebleeding risk after MIS for ICH necessitates a systematic, individualized, and comprehensive intervention strategy encompassing both surgical techniques and patient management.

## Prevention Methods for Postoperative Rebleeding

Preventing postoperative rebleeding after ICH is of paramount importance. Therefore, comprehensive management and strict self-management are necessary to prevent postoperative rebleeding in response to the aforementioned risk factors.

### Preoperative prevention

Preoperative assessment and preparation play a crucial role in preventing postoperative rebleeding following MIS for ICH. Detailed imaging evaluation and rational surgical planning are key steps to ensure surgical success and prevent postoperative rebleeding.

Preoperative imaging assessments can help identify risk factors for postoperative rebleeding. For instance, studies have indicated that preoperative diffusion-weighted imaging (DWI) lesions correlate closely with poor postoperative outcomes, while preoperative cerebral angiography significantly increases the detection rate of DWI lesions [118]. Furthermore, preoperative imaging can help identify hematomas with irregular shapes. In a study, among 116 patients with postoperative rebleeding, 76 (65.52%) showed irregular hematomas on CT, making it an independent predictor of rebleeding [36]. Recognizing these imaging markers enables surgeons to develop more precise surgical plans preoperatively, thereby preventing postoperative rebleeding. Moreover, preoperative assessment and preparation extend beyond imaging to comprehensively evaluate the overall health and functional status of patients. This enables necessary optimization and preparation before surgery, mitigating the risk of postoperative rebleeding [119]. Studies have indicated that preoperative functional status, as measured using the National Institutes of Health Stroke Scale and modified Rankin scale, is closely associated with postoperative functional recovery [24,120]. Therefore, comprehensive preoperative assessment can help physicians predict postoperative outcomes and develop personalized treatment plans.

### Intraoperative prevention

Intraoperative management is also critical in preventing postoperative rebleeding following MIS for ICH. Appropriate hemostasis, blood pressure control, and standardized surgical techniques are critical for surgical success and reducing postoperative rebleeding.

Appropriate intraoperative hemostasis is the key to the prevention of postoperative rebleeding. Studies have indicated that minimally invasive techniques, such as ES, enable effective hematoma evacuation under direct visualization. Intraoperative bleeding can be controlled through irrigation and electrocoagulation, thereby preventing postoperative rebleeding [43,121,122]. Blood pressure management is vital in intraoperative care. Studies have indicated that precise intraoperative blood pressure control helps prevent postoperative rebleeding and improves functional outcomes [123,124]. Similarly, standardized surgical techniques are essential for ensuring surgical safety and efficacy. Minimally invasive surgical techniques are continuously advancing. For example, endoscopic and stereotactic approaches offer less traumatic surgical pathways. Guided by advanced navigation and imaging technologies, standardized surgical techniques by operators can minimize brain tissue injury, thereby preventing postoperative rebleeding [42,43,125,126].

### Postoperative prevention

Postoperative monitoring and management are both indispensable in the prevention of rebleeding after MIS for ICH. Blood pressure management, coagulation monitoring, and imaging follow-up represent core elements of postoperative care, effectively preventing rebleeding events and improving the outcomes of patients.

Strict control of postoperative blood pressure is a critical component in preventing rebleeding after MIS for hematoma evacuation. Although large-scale RCTs specifically addressing blood pressure management after MIS are currently lacking, studies on acute-phase blood pressure reduction in spontaneous ICH provide an important reference. For instance, the Intensive Blood Pressure Reduction in Acute Cerebral Hemorrhage Trial 2 (INTERACT2) study reported that early intensive decrease in blood pressure after MIS improves functional outcomes and may reduce rebleeding risk in patients with extensive ICH [127,128]. This finding suggests that aggressive blood pressure management may also yield benefits in the postoperative phase of MIS. However, postoperative blood pressure targets must be set more individually, fully accounting for the procedure’s impact on cerebral autoregulation. This strategy prevents hypoperfusion of brain tissue due to excessively low blood pressure, which can trigger cerebral hypoxia or metabolic disturbances [129]. Controlling the balance between “rebleeding prevention” and “ensuring cerebral perfusion” is critical in postoperative management. Studies have indicated that reducing mean arterial pressure below 100 mmHg can significantly lower the risk of rebleeding. However, in the postoperative setting, excessively low blood pressure may increase the risk of delayed cerebral ischemia, especially in patients with poor baseline perfusion [130]. Similarly, the Secondary Prevention of Small Subcortical Strokes (SPS3) study confirmed that maintaining the systolic blood pressure of ≤130 mmHg reduces rebleeding risk in patients with ICH and cerebral small vessel diseases [131], providing a reference for long-term blood pressure management in patients with similar conditions undergoing MIS. Therefore, establishing an individualized and rational blood pressure management strategy that effectively prevents rebleeding and fully ensures cerebral perfusion is crucial in the postoperative phases of MIS.

Monitoring coagulation function is as important as blood pressure management. Therefore, postoperative coagulation monitoring and timely correction are paramount for preventing rebleeding [132,133]. Studies have indicated that postoperative coagulation parameters should be monitored immediately and regularly (e.g., every 6 to 12 h), including a complete coagulation panel comprising prothrombin time/international normalized ratio (INR), activated partial thromboplastin time (APTT), platelet count, and fibrinogen level. The therapeutic goal is to correct the INR to <1.5 and maintain APTT within normal ranges to prevent postoperative rebleeding [134,135]. In addition, for patients receiving anticoagulants, the timing of resuming anticoagulation therapy requires careful assessment to balance bleeding and thrombotic risks [136,137].

Similarly, imaging follow-up constitutes another critical component of postoperative management. Early postoperative imaging assessments, particularly cranial CT, can help identify early signs of rebleeding. The “black hole sign” on CT is considered one of the predictors of postoperative rebleeding [138]. Currently, a standardized imaging follow-up is recommended. The standard protocol requires repeated cranial CT within 24 h and at 72 h postoperatively (or whenever clinical status fluctuates) [139,140]. For high-risk patients (e.g., those with large preoperative hematoma, incomplete intraoperative evacuation, or difficult-to-correct coagulation), monitoring frequency should be increased [140]. Once a symptomatic or expanding hematoma is confirmed by imaging, prompt intervention should be initiated to prevent recurrent hemorrhagic events.

**Fig. 5.**
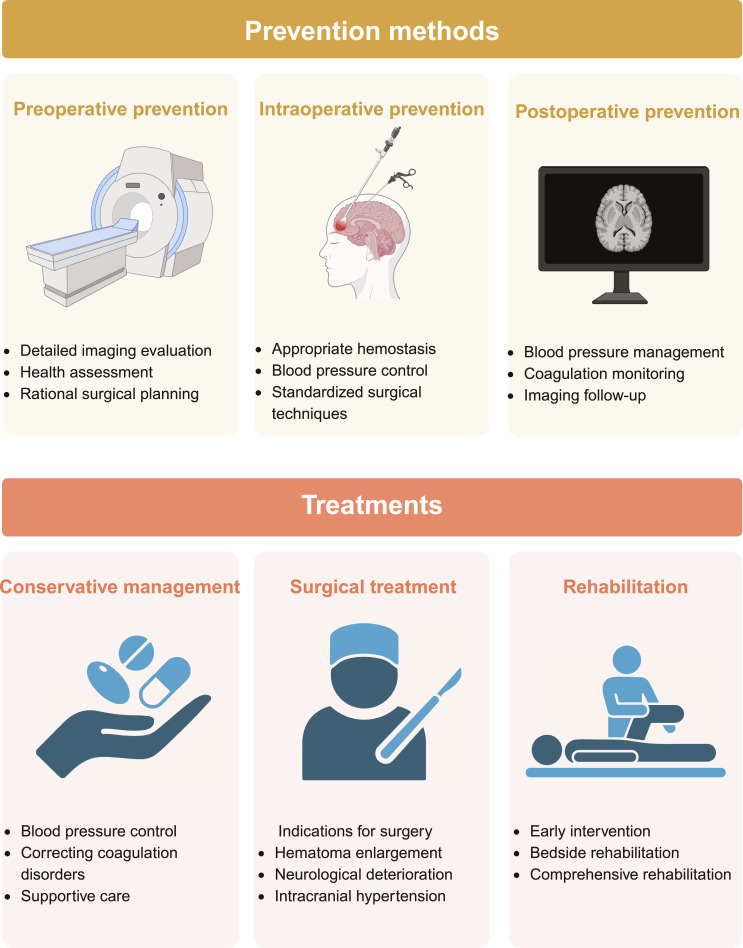
Prevention methods and treatments for postoperative rebleeding. Prevention methods for postoperative rebleeding encompass preoperative, intraoperative, and postoperative prevention. Treatments for postoperative rebleeding include conservative management, surgical treatment, and rehabilitation. Created in BioRender.

In summary, comprehensive measures encompassing preoperative assessment, intraoperative management, and postoperative monitoring can help effectively prevent rebleeding after MIS for ICH, thereby improving patients’ survival and functional prognosis (Fig. 5). Implementation of these measures requires integration with individualized treatment strategies to minimize the incidence of postoperative complications.

## Treatments for Postoperative Rebleeding

Following MIS for ICH, the selection of treatment strategies upon rebleeding requires rapid, comprehensive assessment and decision-making based on the systemic condition of patients, severity of rebleeding, rate of hematoma expansion, and progression of neurological deficits. The core objectives of treatment are to swiftly control active bleeding, reduce the mass effect of the hematoma, prevent secondary brain injury, and establish conditions conducive to neurological recovery. Overall treatment strategies primarily encompass 2 main approaches: conservative management and surgical intervention. Furthermore, rehabilitation therapy should be integrated throughout the entire treatment process (Fig. 5).

### Conservative management

Conservative management refers to treatment approaches other than surgery and serves as the primary and fundamental strategy for managing rebleeding following MIS for ICH. It is particularly suitable when the hematoma is stable and shows no significant enlargement, and the patient’s neurological status remains relatively stable. Its core principle lies in utilizing medical interventions to provide favorable conditions for hemostatic mechanisms, thereby stabilizing the condition and resolving the need for further intervention.

Strict blood pressure control represents the cornerstone of conservative management. Following rebleeding, elevated blood pressure increases the pressure gradient within the hematoma cavity, potentially rupturing the fragile blood crust and leading to active bleeding or hematoma enlargement. Current international guidelines generally recommend maintaining the systolic blood pressure below 130 to 140 mmHg during the acute phase of rebleeding as a relatively safe and effective target [141–143]. Blood pressure reduction must be gradual and in a controlled manner to prevent sudden drops that may lead to cerebral hypoperfusion and secondary ischemic injury [144]. Short-acting intravenous antihypertensive agents (e.g., urapidil and nicardipine) are typically preferred because of real-time monitoring and precise dose adjustment [145,146].

In addition to strictly controlling blood pressure, it is critical to correct existing coagulation disorders. For patients receiving anticoagulants or antiplatelet agents, immediate reversal measures should be initiated. Those receiving anticoagulants (e.g., warfarin and DOACs) should urgently receive specific reversal agents (e.g., vitamin K + prothrombin complex concentrate (PCC)/fresh frozen plasma (FFP), idarucizumab, and andexanet alfa) [147,148]. Platelet transfusion or desmopressin may be administered for patients receiving antiplatelet therapy [149]. Beyond medications correcting coagulation disorders, hemostatic agents are also clinically important. Theoretically, hemostatic agents, such as recombinant activated factor VII (rFVIIa) and tranexamic acid, can enhance the hemostatic process. However, current clinical guidelines no longer routinely recommend their use for treating spontaneous ICH (including rebleeding). This is primarily due to their unfavorable risk–benefit ratio. Several large RCTs (e.g., the Factor Seven for Acute Hemorrhagic Stroke (FAST) trial) have shown that although rFVIIa reduces the risk of hematoma expansion, it fails to improve long-term neurological outcomes or mortality rates [150]. More concerning, it significantly increases the risk of arterial thromboembolic events, such as myocardial infarction and cerebral infarction [151]. Therefore, except for extremely specific circumstances with a clear coagulation factor deficiency, hemostatic agents must be used with extreme caution.

Similarly, comprehensive supportive care forms the foundation for stabilizing the patient’s condition. Supportive care includes maintaining electrolyte balance, providing nutritional support, and controlling body temperature. It also involves actively treating fever to reduce cerebral metabolic rate, closely monitoring ICP with administration of agents like mannitol or hypertonic saline when necessary, and preventing complications such as deep vein thrombosis, stress ulcers, seizures, and pulmonary infections [152–154].

Glucocorticoids, as potent anti-inflammatory agents, theoretically reduce cerebral edema and secondary injury by suppressing inflammatory responses. However, their use in spontaneous ICH remains controversial. The Chinese Guidelines for the Diagnosis and Treatment of Intracerebral Hemorrhage state that glucocorticoids are commonly administered to prevent cellular edema through anti-inflammatory effects; however, they offer no significant benefit in patients with spontaneous ICH and increase the risk of complications, such as infection, gastrointestinal bleeding, and hyperglycemia. Therefore, glucocorticoids should not be routinely administered to patients with ICH. The AHA/ASA guidelines explicitly state that glucocorticoids should not be administered to improve outcomes in patients with ICH, as they provide no clinical benefits and may increase the risk of complications [155]. These risks are particularly notable in patients with ICH, as hyperglycemia and infection can exacerbate secondary brain injury, prolong hospital stays, and increase the risk of rebleeding. Thus, in routine clinical practice, glucocorticoids should not be considered standard anti-inflammatory therapy for ICH. MIS, as an invasive procedure, triggers a local traumatic response in ICH. It disrupts the blood–brain barrier and induces inflammatory cell infiltration and cytokine release, thereby exacerbating cerebral edema and the inflammatory cascade [156]. Theoretically, surgery-induced inflammation may hinder neurological recovery and increase the risk of rebleeding. In such scenarios, the anti-inflammatory properties of glucocorticoids could be considered to control excessive inflammatory responses, but their application must be strictly confined to specific contexts. Following MIS, neurosurgeons may consider short-term, localized, or systemic glucocorticoid therapy under the following circumstances: (a) alleviating significant vasogenic edema around the surgical pathway [157]: If postoperative imaging reveals severe edema around the surgical pathway or hematoma causing mass effect and increased ICP, short-term glucocorticoids (e.g., dexamethasone administered locally via drainage tubes) may be used to reduce edema and lower the risk of rebleeding; (b) managing severe mass effect secondary to hematoma: When hematoma leads to significant mass effect and standard measures (e.g., mannitol) prove ineffective, glucocorticoids may serve as adjunctive therapy to control inflammation-mediated swelling [158]. While these delivery methods (e.g., local administration) can theoretically prevent systemic side effects, prospective studies confirming their safety are currently lacking. Systemic administration should be limited to short courses with close monitoring for complications. Currently, no high-level evidence (e.g., RCTs or meta-analyses) supports the routine use of glucocorticoids after MIS for ICH. Future studies are needed to explore local administration or targeted anti-inflammatory strategies that minimize risks while controlling inflammation. Currently, the effects of nonsteroidal anti-inflammatory drugs, particularly aspirin, have been explored. The 2019 REstart or STop Antithrombotics Randomised Trial (RESTART) study found that aspirin therapy after ICH did not increase the risk of recurrent bleeding during a 2-year follow-up period and showed a trend toward reducing rebleeding, potentially related to aspirin’s anti-inflammatory effects [159]. The 2024 E-start trial demonstrated that patients with spontaneous ICH who received early aspirin therapy postoperatively had a lower rate of rebleeding compared to those who received aspirin later. While the primary mechanism for this observation may be attributed to aspirin’s antiplatelet effects in preventing microthrombus formation and improving local microcirculation, its potent anti-inflammatory properties may also exert effects by modulating the postoperative inflammatory environment [160]. These findings indirectly support the concept that mitigating the inflammatory response following MIS may create a more stable environment conducive to hemostasis and reduced rebleeding risk. Naturally, these studies carry limitations: The RESTART study did not exclusively include patients undergoing MIS; in the E-start study, safety outcomes may have lacked sufficient statistical power due to the low number of postoperative bleeding events (5 total). Currently, there is no high-level evidence from RCTs supporting the routine use of aspirin or other nonsteroidal anti-inflammatory drugs specifically for preventing rebleeding after spontaneous ICH following MIS. In this unique postoperative setting, future prospective studies are needed to explore the safety and efficacy of targeted short-term anti-inflammatory regimens (including but not limited to aspirin, as well as potentially safer alternatives).

Currently, active management of sympathetic hyperactivity induced by central nervous system injury (commonly termed “sympathetic storm”) is gaining increasing attention in the intensive care of ICH (including post-MIS rebleeding). This state of autonomic dysfunction is characterized by intermittent or sustained catecholamine surges, leading to blood pressure instability, tachycardia, tachypnea, hyperthermia, profuse sweating, and dystonia [161]. In rebleeding scenarios, sympathetic storms may exacerbate cardiovascular burden, increase cerebral metabolic demands, promote systemic inflammatory responses, and directly trigger hematoma instability by causing marked fluctuations in cerebral perfusion pressure. Therefore, neuromodulation strategies aimed at alleviating this adrenergic hyperactivity represent a crucial adjunct to conventional blood pressure management. Primary pharmacological approaches involve centrally or peripherally acting sympatholytic agents. Beta-blockers (e.g., propranolol and labetalol) serve as foundational medications [162]. These drugs effectively alleviate symptoms and improve patient outcomes by suppressing excessive sympathetic nervous system activation. Future research should further explore combination therapies and their efficacy across diverse patient populations to optimize treatment protocols.

### Surgical treatment

Surgical intervention should be promptly considered when conservative management cannot prevent disease progression, and rebleeding leads to progressive hematoma expansion, causing significant mass effect and worsening neurological deficits [163]. As a salvage procedure, surgery aims to evacuate the hematoma, effectively reduce ICP, relieve brainstem compression, and provide an opportunity for thorough hemostasis under direct visualization.

Surgical intervention must strictly adhere to indications. The most critical criterion is significant hematoma enlargement, accompanied by a marked mass effect. Specifically, reoperation should be actively considered when supratentorial hematomas exceed 30 ml (especially in superficial locations) or infratentorial hematomas exceed 10 ml, leading to a midline shift of >5 mm, ventricular compression, or acute hydrocephalus, especially if the patient’s condition progressively deteriorates [164,165]. Concurrently, when rebleeding leads to progressive neurological deterioration, which manifests as a persistent decline in consciousness (e.g., Glasgow Coma Scale (GCS) score drop ≥2 points), dilated pupils, or new focal neurological deficits, and imaging confirms a close association with hematoma expansion, urgent decompressive surgery is necessary to decrease the risk of brain herniation [166,167]. Furthermore, when intracranial hypertension remains uncontrolled despite standardized conservative management, including dehydration therapy, sedation, and hyperventilation, particularly with a persistent ICP of >25 mmHg attributable to rebleeding hematoma, reoperation becomes an unavoidable but necessary option [168,169]. However, for elderly patients with multiple comorbidities or severe brainstem dysfunction or for those already in a moribund state preoperatively, surgical intervention offers minimal benefit and is generally not recommended.

Therefore, the decision for reoperation following rebleeding after MIS for ICH must be swift and prudent. This means that the decision must be made on the basis of immediate noncontrast cranial CT imaging to rapidly confirm the diagnosis and extent of rebleeding and after a comprehensive assessment of the patient’s overall condition and prognosis. Regarding surgical approaches, options such as reentry via the original access route, craniotomy with hematoma evacuation, or decompressive craniectomy can be selected on the basis of the circumstances [170,171]. Compared to the initial surgery, reoperation significantly increases technical difficulty and risk. Thus, it must be conducted by experienced specialists to minimize complications and improve treatment outcomes.

### Rehabilitation

Rehabilitation therapy is not merely a supplementary measure following the acute phase but an integral component of the overall treatment system, requiring early intervention and continuous implementation throughout the entire process. Rebleeding offers a “secondary blow” to the central nervous system, making systematic rehabilitation crucial for functional recovery. Therefore, once a patient’s vital signs stabilize, an individualized rehabilitation plan should be initiated and advanced in a phased and focused manner [172]. First, bedside rehabilitation should commence during the ultra-early phase (in the intensive care unit or stroke unit). Through measures such as optimal positioning, passive range-of-motion exercises, and respiratory management, this phase prevents complications, such as contractures, pressure ulcers, aspiration pneumonia, and deep vein thrombosis, while preserving joint mobility and cardiopulmonary reserve for subsequent active training. Subsequently, with the improvement in patients’ condition and stabilization of the intracranial status, a gradual transition to a multimodal comprehensive rehabilitation phase is necessary. This includes progressively intensifying physical therapy, occupational therapy, speech and swallowing therapy, cognitive function training, and necessary psychological interventions. Such modifications aim to maximize neurological recovery and improve patient prognosis [173–175]. In summary, rehabilitation following rebleeding should adhere to the principle of “early intervention and comprehensive implementation”. Through multimodal, multidisciplinary collaboration, this strategy promotes neural functional remodeling and compensation while significantly improving the quality of life of patients in the long term.

In conclusion, managing rebleeding after MIS for ICH is a dynamic, multidisciplinary, and tiered process. It relies on 3 interconnected pillars: (a) meticulous conservative management as the foundation, (b) surgically indicated interventions as the critical intervention, and (c) early, systematic rehabilitation as the functional recovery safeguard. Only such an integrated approach can optimize the prognosis of patients with this critical complication.

## Discussion and Outlook

This article provides a systematic overview of the definition, mechanisms, risk factors, prevention, and treatment strategies for rebleeding following MIS for ICH. In summary, rebleeding represents one of the most severe complications following MIS for ICH. It originates from the complex interplay of 3 factors: the patient’s inherent vascular fragility (e.g., hypertensive vascular disease), the pathophysiological effects of the hematoma itself (e.g., inflammation and oxidative stress), and the manipulations inherent to MIS (e.g., decompressive injury and mechanical stimulation). Although MIS introduces significantly less surgical trauma compared to traditional craniotomy, the risk of postoperative rebleeding remains substantial and is closely associated with poor neurological outcomes and mortality. The current multidimensional strategy for preventing rebleeding includes the following: (a) identification and management of patient-related factors, such as hypertension, coagulation disorders, deep hemorrhagic sites, and large hematoma volume; (b) identification and management of surgery-related factors, such as careful timing and technique selection; and (c) enhancing surgical proficiency. Once rebleeding occurs, it is necessary to make rapid decisions based on hematoma stability, mass effect, and neurological status. This involves either conservative medical management focused on aggressive blood pressure control and reversal of coagulation or surgical intervention through emergency decompressive surgery as a life-saving measure, supplemented by early, systematic rehabilitation therapy.

**Fig. 6.**
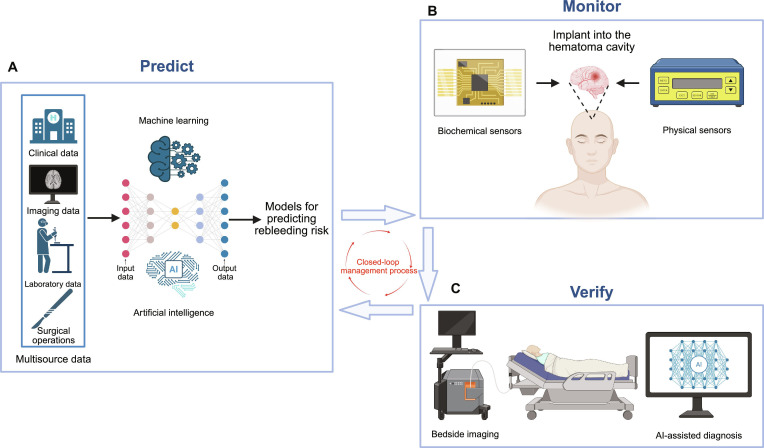
An ideal real-time monitoring system for postoperative rebleeding. (A) By integrating ML and AI technologies to consolidate multisource data from clinical, imaging, laboratory, and surgical operations to construct dynamic, individualized models for predicting rebleeding risk. (B) Developing microsensors (biochemical and physical sensors) implantable within hematoma cavities to enable continuous monitoring. (C) Bedside imaging technologies and AI-assisted diagnosis. This forms a complete predict-monitor-verify closed-loop management process. Created in BioRender.

However, despite the identification of risk factors and implementation of preventive strategies, rebleeding persists in clinical practice because the monitoring and diagnosis of postoperative rebleeding heavily rely on CT. For critically ill patients, routine daily CT follow-ups serve as a vital tool for monitoring, enabling early warning or confirmation of rebleeding through the identification of imaging markers, such as the “blend sign” [176,177]. As the diagnostic gold standard, CT provides definitive anatomical evidence. However, this monitoring is inherently point in time and cannot achieve real-time continuous surveillance. Therefore, a monitoring paradigm capable of delivering continuous, proactive bedside alerts that precede imaging changes is necessary for clinical practice. Future monitoring models for postoperative rebleeding in ICH should transition from the current “lagging and passive” approach toward a multidimensional, continuous, closed-loop early warning system characterized by “real-time, proactive, and intelligent” capabilities. To achieve this goal, studies should focus on integrating machine learning (ML) and artificial intelligence (AI) technologies. This involves consolidating multisource data from clinical, imaging, laboratory, and surgical operations to construct dynamic and individualized models for predicting rebleeding risk. By incorporating continuous data streams, such as real-time blood pressure variability and dynamic changes in coagulation function, risk levels can be updated in a real-time manner. This enables precise identification of high-risk patients and can help optimize their monitoring strategies. For example, Hall et al. [178] developed and validated an ML model based on data from patients with ICH. This model integrated multidimensional features, including hematoma volume, medication history, demographics, and pre-ICH modified Rankin scale scores. The results showed that its area under the curve for predicting rebleeding was significantly higher than traditional scoring systems [178]. Furthermore, ML models exhibited strong performance in predicting in-hospital mortality from ICH, with significantly enhanced predictive accuracy when combining clinical and imaging data [179]. These studies highlight the substantial advantages of AI models in integrating complex, nonlinear clinical data, providing a viable tool for achieving precise and individualized risk stratification. Simultaneously, this strategy establishes a crucial foundation for future development of personalized models predicting the risk of rebleeding. Future studies should also develop microsensors implantable within hematoma cavities. For instance, biochemical sensors can allow continuous monitoring of hemoglobin concentration, partial pressure of oxygen, pH, or specific biomarkers (e.g., D-dimer) in the drainage fluid or interstitial fluid, acting as “chemical alarms” to detect early-stage bleeding. Integration of physical sensors, which can help simultaneously monitor ICP and Doppler blood flow signals, can enable dual validation through “physical monitoring + blood flow assessment”. Miniaturization and integration represent key trends in the ongoing advancement of sensor technology. Miniature sensors, such as one-dimensional implantable sensors, enable real-time, precise monitoring of physiological and pathological parameters with minimal invasiveness [180]. Simultaneously, sensors integrating multiple sensing functions, such as those combining pressure, temperature, and flow rate monitoring, offer more comprehensive surveillance [181]. The injectable, bioabsorbable, and wireless meta-structured hydrogel (metagel) sensor reported by Tang et al. [182] represents a cutting-edge technology for ultrasound monitoring of intracranial signals. Implanted into the intracranial space with a puncture needle, the metagel deforms in response to changes in the physiological environment, causing peak frequency shifts of reflected ultrasound waves that can be wirelessly measured using an external ultrasound probe. The metagel sensor can independently detect ICP, temperature, pH, and flow rate, providing a detection depth of 10 cm and fully degrading within 18 weeks [182]. These studies demonstrate the flexibility, biodegradability, and high precision of miniaturized sensors, which perfectly meet the need for short-term, continuous, and safe monitoring of hematoma following MIS for brain hemorrhage. They point the way toward developing next-generation in vivo postoperative monitoring systems. Innovations in bedside imaging technologies and AI-assisted diagnosis are equally critical. Contrast-enhanced ultrasound (CEUS), performed at the bedside, can indicate active bleeding by detecting contrast agent “extravasation” and holds promise as a routine postoperative screening tool. In addition, deep-learning-based algorithms should be developed to enable automatic registration, segmentation, and comparison of continuous CT images, sensitively identifying microhematoma changes even smaller than 5 ml. Automated alert mechanisms can significantly enhance diagnostic efficiency and sensitivity. In summary, an ideal real-time monitoring system for postoperative rebleeding in ICH should be AI-driven, encompassing dynamic risk prediction before and after surgery. It should guide the implantation of in-body sensors and continuous monitoring for high-risk patients, rapidly initiating CEUS verification after detecting abnormalities. This forms a complete “predict-monitor-verify” closed-loop management process. Such a system holds promise for achieving early diagnosis and management of rebleeding (Fig. 6). In the closed-loop management envisioned by this study, AI-driven dynamic prediction and in vivo sensor monitoring form a complementary, organic whole with routine CT examinations. The AI system acts as an ever-vigilant “physiological radar”, continuously identifying high-risk patients. Upon detecting escalating risks or abnormal signals, it immediately triggers targeted, advanced examinations, such as CEUS or CT. This model optimizes imaging resources from “fixed-schedule screening” to “intelligent precision validation”. It preserves the authority of CT in anatomical diagnosis while endowing the entire monitoring system with unprecedented continuity and foresight. Thus, this system can help evolve from “periodic snapshots” to “continuous surveillance + intelligent shutter”.

Although the multidimensional closed-loop early warning system outlined in this paper holds great promise, its transition from concept to widespread clinical application faces a series of technical and practical challenges that should be addressed in future studies. First, at the data and AI model level, the multisource data required to build dynamic prediction models exist across heterogeneous systems with inconsistent formats, forming “data silos” [183]. Developing unified data standards and cross-platform integration protocols is a primary prerequisite. Simultaneously, models trained on single-center data often suffer from insufficient generalization due to variations in patient populations and clinical workflows. Their “black-box” decision-making logic also undermines clinical trust, necessitating multicenter collaboration and explainable AI technologies to enhance model universality and transparency. Second, regarding microbiosensors, their implantation faces rigorous challenges in terms of biocompatibility, long-term safety, and signal stability. Protein adsorption and tissue encapsulation in the in vivo environment can cause signal drift [184]. Developing anticontamination materials, in vivo self-calibration algorithms, and solutions for energy supply and wireless data transmission during long-term implantation are core challenges to ensuring the reliability of monitoring. Furthermore, at the clinical integration and regulatory level, the system must be seamlessly embedded into existing workflows, and high signal-to-noise ratio alerts must be utilized to avoid burdening healthcare providers. Simultaneously, it must undergo rigorous approval by regulatory authorities and provide robust health economic data supporting its safety, efficacy, and cost-effectiveness. In summary, interdisciplinary “medicine-engineering-information technology” teamwork is needed to collectively tackle these challenges and achieve iterative technological optimization and rigorous clinical trials to transform this “real-time, proactive, intelligent” monitoring concept into a practical tool. This will finally establish a robust safety barrier for patients recovering from ICH.

## Data Availability

This review is based on previously published studies. No new data were generated or analyzed in this study. All referenced data can be accessed through the cited publications.
